# Management of sacroiliac joint pain: current concepts

**DOI:** 10.1007/s00590-025-04308-2

**Published:** 2025-05-21

**Authors:** Filippo Migliorini, Ludovico Lucenti, Tommaso Bardazzi, Andreas Bell, Federico Cocconi, Nicola Maffulli

**Affiliations:** 1 Department of Trauma and Reconstructive Surgery, University Hospital in Halle, Halle, Germany; 2 Department of Orthopaedic and Trauma Surgery, Academic Hospital of Bolzano, Bolzano, Italy; 3 Department of Precision Medicine in Medical, Surgical and Critical Care (Me.Pre.C.C.), University of Palermo, Palermo, Italy; 4 Department of Trauma and Orthopaedic Surgery, Eifelklinik St.Brigida, Simmerath, Germany; 5 Faculty of Medicine and Psychology, Sapienza University of Rome, Rome, Italy; 6 Centre for Sports and Exercise Medicine, Barts and the London School of Medicine and Dentistry, Mile End Hospital, Queen Mary University of London, London, UK; 7 School of Pharmacy and Bioengineering, Keele University Faculty of Medicine, Stoke on Trent, United Kingdom; 8 Department of Life Sciences, Health, and Health Professions, Link Campus University of Rome, Rome, Italy

**Keywords:** Sacroiliac, Sacroiliitis, Pain, Management

## Abstract

**Introduction:**

Managing sacroiliac joint (SIJ) pain is challenging and unpredictable. There are no internationally accepted recommendations. In light of the lack of global consensus and guidelines and the ongoing advancements in management options, a widely accepted treatment algorithm remains absent. This systematic review updates and evaluates the existing evidence on strategies for managing SIJ pain.

**Methods:**

This study followed the guidelines defined in the 2020 PRISMA statement. All clinical studies concerning the clinical management of SIJ pain were considered. Web of Science, PubMed, and Embase were accessed in January 2025 without additional filters or temporal constraints. The risk of bias evaluation and statistical analysis followed the guidelines described in the Cochrane Handbook for Systematic Reviews of Interventions.

**Results:**

Fifteen randomised controlled trials, 13 clinical trials, and 10 retrospective studies were included. Data from 2666 patients (1429 women) were retrieved. The mean length of follow-up was 14.7 ± 15.2 months. The mean age was 54.0 ± 5.8 years, and the mean BMI was 28.5 ± 2.5 kg/m^2^. Non-surgical options primarily focus on physical therapy to relieve discomfort. Different medications aim to decrease inflammation and pain at the SIJ. Fluoroscopically guided SIJ injections allow for directly administering steroids or mesenchymal stem cells into the joint. Radiofrequency denervation is frequently used to address SIJ pain, while surgical fusion is usually reserved for cases where conservative treatment is ineffective.

**Conclusion:**

Managing SIJ pain is challenging due to limited and inconsistent evidence. Treatment progresses from conservative options, physiotherapy, lifestyle changes, and non-steroidal anti-inflammatory drugs to more invasive approaches like injections, radiofrequency denervation, and, in severe cases, surgical management. Research limitations include small sample sizes, short follow-ups, and inconsistent methodologies. Future high-quality studies are needed to establish clear diagnostic and treatment guidelines, compare techniques, and explore new therapies like regenerative medicine.

**Supplementary Information:**

The online version contains supplementary material available at 10.1007/s00590-025-04308-2.

## Introduction

Sacroiliac (SIJ) pain is common [1–4]. SIJ pain is reproducible by stress and provocation tests of the SI joint, and selective injection with a local anaesthetic promotes symptom remission [5]. SIJ contributes to 15% to 30% of low back pain [6, 7], representing a global healthcare issue that burdens health systems, with an estimated three-year cost of $1.6 billion per 100,000 people for conservative management alone [8]. The SIJ interacts synergistically with the hip due to the extensive connections of muscles, tendons, and ligaments [9, 10]. The causes of SIJ pain are either intraarticular (infection, arthritis, spondyloarthropathies, malignancies) or extra-articular (enthesopathy, fractures, ligamentous injuries, and myofascial inflammation) [11–14]. Potential risk factors for SIJ pain are leg length discrepancy, trauma, scoliosis, lumbar fusion surgery with fixation of the sacrum, heavy physical exertion, and pregnancy [15–24]. Given the anatomical complexity of the involved region and the various and unclear pain manifestations, the diagnosis of SIJ dysfunction is challenging [12, 14, 25]. Therefore, a comprehensive clinical and imaging evaluation is needed. Pain is generally located in the lower lumbar region (72%), groin (14%), upper lumbar region (6%), or abdomen (2%). Pain referred to as the lower limb occurs in 28% of patients, and 12% also report foot pain [2, 26, 27]. Physical examinations, such as palpation and tenderness in the sacral region, are specific and reliable in the right hands [27–31]. Other tests, such as Trendelenburg and gait assessment, may be misleading, having poor specificity in detecting SIJ pain. Positivity at abduction and extra-rotation (ABER test), Gaenslen’s, distraction, thigh thrust, and compression tests have good sensitivity in detecting SI joint pain [27, 32, 33]. Historically, ultrasound-guided injection of local anaesthetic drugs in the SIJ represents the gold standard for diagnosis [34, 35].

The management of SIJ pain is challenging and unpredictable, with internationally accepted recommendations missing. Given the lack of international consensus and guidelines, a widely accepted treatment algorithm remains elusive alongside the continuous advancement of surgical and non-surgical management options [36–41]. This systematic review updates and discusses the current evidence regarding non-interventional and interventional strategies for managing SIJ pain.

## Methods

### Eligibility criteria

The present systematic review followed the guidelines defined in the 2020 Preferred Reporting Items for Systematic Reviews and Meta-Analyses (PRISMA) statement [42]. All clinical studies concerning the clinical management of SIJ pain were considered. Eligible studies were required to be published in peer-reviewed journals. Only articles in the following languages were included: English, German, Italian, French, or Spanish. Only studies classified as levels I to IV of evidence were included, according to the 2020 Oxford Centre of Evidence-Based Medicine [43].

### Search strategy

The literature search followed the reported algorithm:

- Problem: SIJ pain;
- Intervention: conservative and surgical management;
- Outcomes: PROMs, complications;
- Design: clinical study.

Web of Science, PubMed, and Embase were accessed in January 2025 without additional filters or temporal constraints. The Medical Subject Headings (MeSH) utilised in the database search are noted in Appendix.

### Selection and data collection

Two authors (FM and FC) independently conducted the database search. All the titles underwent manual screening, and their abstracts were reviewed if deemed relevant. Full texts were scrutinised for the articles matching the inclusion criteria. Articles lacking accessible full texts were excluded. Furthermore, a cross-reference of the bibliographies of full-text articles was performed for potential inclusion. Any discrepancies between authors were resolved by a third senior author (NM), who made the final decision.

### Data items

Two authors (FM and FC) independently conducted data extraction. The following generalities were collected for each study: the name of the first author, the year and journal of the publication, the design of the study, and the mean length of follow-up (months). The following data at baseline were extracted: the number of patients and women and the mean body mass index (BMI).

### Assessment of the risk of bias

The risk of bias evaluation followed the guidelines described in the Cochrane Handbook for Systematic Reviews of Interventions [44]. The risk of bias in the selected articles was independently assessed by two authors (FM and TB). Randomised controlled trials (RCTs) were checked against the revised risk of bias assessment tool (RoB2) [45, 46] of the Cochrane tool for assessing the risk of bias in randomised trials (RoB) [47]. The following endpoints were considered: bias arising from the randomisation process, bias due to deviations from intended interventions, bias due to missing outcome data, bias in the measurement of the outcome, and bias in the selection of the reported result. To analyse the risk of bias in non-randomised controlled trials (non-RCTs), the Risk of Bias in Nonrandomised Studies of Interventions (ROBINS-I) tool [48] was utilised. The tool considers seven domains of potential bias. These domains include confounding factors and patient selection characteristics before the comparative intervention, bias in classification during the intervention, methodological quality post-intervention comparison, which involves deviations from intended interventions, missing data, inaccurate outcome measurement, and bias in reported outcome selection. The chart of the ROBINS-I was generated using the Robvis Software (Risk-of-bias VISualization, Riskofbias.info, Bristol, UK) [49].

### Synthesis method

All statistical analyses were performed by the main author (XX) following the recommendations of the Cochrane Handbook for Systematic Reviews of Interventions [44]. Descriptive statistics were calculated using the IBM SPSS software version 25 (International Business Machines Corporation, Armonk, USA). The arithmetic mean and standard deviation were used for continuous data, and the frequency (events/observations) is used for dichotomic variables.

## Results

### Study selection

**Fig. 1:**
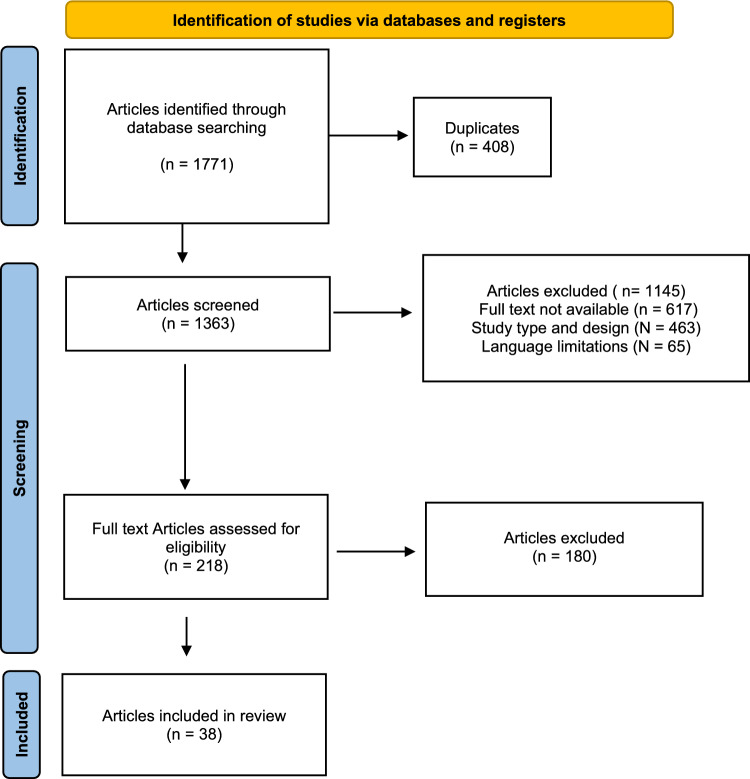
PRISMA flow chart of the literature search

The systematic literature search resulted in the identification of 1771 articles. After removing duplicates, the abstracts of 1363 articles were screened for eligibility. A total of 1145 articles were excluded for the following reasons: mismatch with the predefined study design criteria (N = 463), full-text unavailability (N = 617), and language limitations (N = 65). Of the remaining 218 studies, another 180 were excluded after full-text evaluation. Consequently, 38 studies were included in this systematic review. The results of the literature search are shown in Fig. 1.

### Risk of bias assessment

**Fig. 2:**
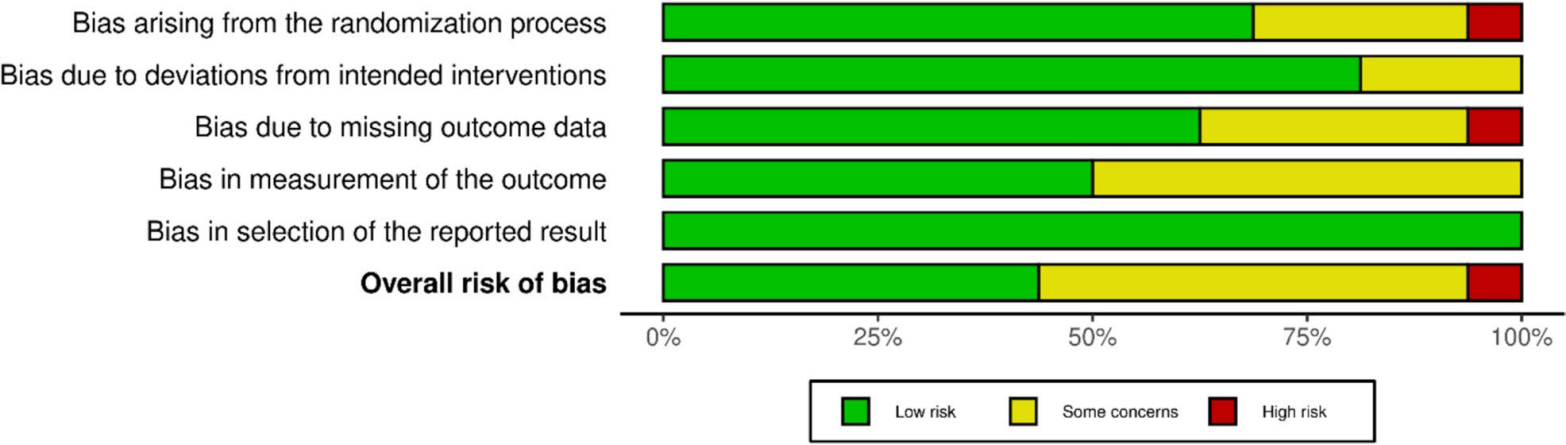
Cochrane risk of bias 2.0 tool (RoB2 tool)

The Cochrane Risk of Bias Assessment tool (RoB2) was applied to evaluate the 15 randomised controlled trials (RCTs) out of the 38 included articles (40%). Analysis revealed a low or moderate risk of bias introduced by the randomisation process in nearly all the included RCTs. Deviations from the intended intervention resulted in a low risk of bias for 80% of the studies, with the remaining studies exhibiting a moderate risk. Only one RCT demonstrated a high risk of bias due to missing data, while the remaining studies were judged to have a low or moderate risk. The risk of bias in outcome measurement was low in half of the included studies. At the same time, some concerns emerged in the remaining studies, primarily from a lack of blinding for outcome assessors. Lastly, all studies demonstrated a low risk of bias in selecting reported results. In conclusion, the RoB2 assessment indicated a moderate or low risk of bias for all but one study, suggesting an acceptable level of methodological quality across the RCTs (Fig. 2).

**Fig. 3:**
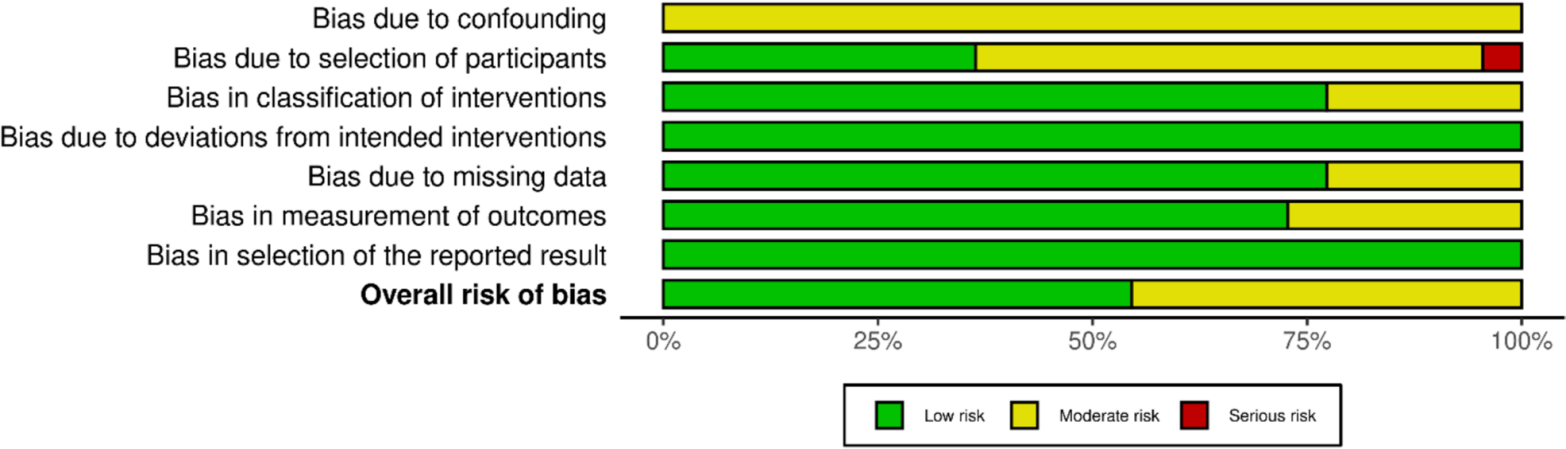
ROBINS-I of non-RCTs

The ROBINS-I tool was employed to evaluate the risk of bias in the selected non-RCTs (22 of 38 articles). All articles exhibited a moderate risk of bias in the first domain. This is a key limitation in the methodological quality of the included studies. The risk of bias arising from participant selection was judged low for approximately one-third of the articles, with a moderate risk observed in nearly all others. Only one article demonstrated a serious risk of bias in this domain. Encouragingly, three-quarters of the articles exhibited a low risk of bias in domains 3, 5, and 6, which assess the risk of bias due to classification of interventions, missing data, and outcome measurement, respectively. Furthermore, all articles demonstrated a low risk of bias due to deviation from the intended intervention and selection of the reported result. In conclusion, the ROBINS-I assessment indicated a low to moderate overall risk of bias for all included non-RCTs, suggesting an acceptable level of methodological quality with the caveat of potential bias in the first domain (Fig. 3).

### Study characteristics and results of individual studies

Data from 2666 patients were retrieved, 1429 of whom were women. The mean length of follow-up was 14.7 ± 15.2 months. The mean age was 54.0 ± 5.8 years, and the mean BMI was 28.5 ± 2.5 kg/m^2^. The generalities of the included studies are shown in Table 1.

**Table 1:** Generalities of the included studies

Author and year	Journal	Design	Follow-up (months)	Patients (n)	Women (n)	Mean age (y)	Mean BMI
Alqhtani et al. 2023 [50]	*Life(basel)*	RCT	6.0	120	60	45.0	
Atluri et al. 2022 [51]	*Pain Physician*	Prospective	12.0	80		60.0	32.9
Bayerl et al. 2020 [52]	*Neurosurg Rev*	Retrospective	12.0	121	88	59.1	
Bertoldo et al. 2021 [53]	*Medicina (Kaunas)*	Prospective	1.0	20	4	46.6	24.8
Brennick et al. 2021 [54]	*Pain Physician*	Prospective	12.0	14	10	33.0	32.7
Burhman et al. 2007 [55]	*Reg Anaesth pain med*	Prospective					
Cahueque et al. 2023 [56]	*N Am Spine Soc J*	Retrospective	12.0	45	17	62.6	
Chauhan et al. 2019 [57]	*Neurospine*	RCT		29			31.0
Chen et al. 2023 [58]	*Neurospine*	Retrospective	12.0	72	51	63.0	26.0
Cheng et al. 2013 [59]	*Clin J Pain*	Retrospective	12.0	88			
Cheng et al. 2016 [60]	*Pain Physician*	Prospective	12.0	93	71	53.5	29.0
Cohen et al. 2003 [61]	*Reg Anaesth pain med*	Prospective		18	10		
Cohen et al. 2023 [62]	*Reg Anaesth pain med*	RCT	3.0	210	145	56.8	28.9
Dengler et al. 2016 [63]	*Acta Neurochir (wien)*	RCT	6.0	101	74	46.8	
Dengler et al. 2019 [64]	*J Bone Joint Am*	RCT	24.0	103	75	48.0	27.0
Ding et al. 2018 [65]	*J Pain Res*	Retrospective	12.0	64	24	50.0	
Dutta et al. 2018 [66]	*Pain Physician*	RCT	6.0	30	21	42.3	
Fuks et al. 2018 [67]	*Eur Spine J*	Prospective	24.0	171	115	54.0	
Gaetani et al. 2013 [68]	*J Neurosurgery Sci*	Prospective	18.0	12	12	53.0	
Haufe et al. 2005 [69]	*Photomed Laser Surg*	Prospective	12.0	38			
Ho et al. 2013 [70]	*J Pain Res*	Prospective	24.0	20			
Jee et al. 2014 [71]	*Arch Phys Med Rehabil*	RCT	3.0	120	89	60.8	23.3
Kleinmann et al. 2020 [72]	*Scand J Pain*	Observational	15.4	22	14	65.8	
Lynch et al. 2022 [73]	*Ortho Res Rev*	Prospective	6.0	57		63.0	
Metha et al. 2018 [74]	*Pain Physician*	RCT	6.0	17		58.5	
Patel et al. 2012 [75]	*Pain Med*	RCT	9.0	51	37	58.0	
Patel et al. 2017 [76]	*Pain Pract*	RCT	12.0	51			
Polly et al. 2015 [77]	*Neurosurgery*	RCT	12.0	158	102	50.9	30.3
Polly et al. 2016 [78]	*Int J Spine Surg*	RCT	24.0	148	102	50.9	30.3
Romero et al. 2015 [79]	*Arq Neuropsichiatr*	Prospective	18.0	32	14	58.3	
Rudolph et al. 2013 [80]	*Open Orthop J*	Retrospective	24.0	40	26	56.0	
Srejic et al. 1999 [81]	*Reg Anaesth pain med*	Case Report		4			
Sturesson et al. 2017 [82]	*Eur Spine J*	RCT	6.0	103	75	48.1	27.0
Sulemain et al. 2017 [83]	*Ghana Med J*	Prospective	12.0	26	10	57.5	
Tinnirello et al. 2017 [84]	*Pain Med*	Retrospective	12.0	43	31	60.9	23.7
van Tilburg et al. 2016 [85]	*Clin J Pain*	RCT	3.0	60	50	60.0	28.0
Vanaclocha et al. 2018 [86]	*Neurosurgery*	Retrospective	72.0	137			
Whang et al. 2015 [87]	*Int J Spine Surg*	RCT	6.0	148	102	51.0	30.4

## Discussion

The management of SIJ pain is multidisciplinary, involving pain management specialists, rheumatologists, orthopaedic surgeons, and physical medicine and rehabilitation physicians. It includes different methods according to patient conditions and requests [88, 89]. The best method to diagnose and manage SIJ pain is continuously discussed [36–40, 90]. Several factors, including the complexity of the pathology, the heterogeneity in aetiologies, the variability in patient presentations, the challenges associated with diagnosis, and the development of treatment modalities, impair the standardisation of managing this condition [26, 30, 91, 92].

SIJ pain is multifactorial, including infections, psoriatic arthritis, and ankylosing spondylitis [33, 93, 94]. Depending on the underlying cause, SIJ pain can have different pathophysiologies, clinical presentations, and outcomes, frequently necessitating individualised treatment plans [35, 95]. Symptoms of SIJ pain vary largely between modest discomfort and severe pain [30, 96–100]. While some individuals may benefit from conservative management, such as NSAIDs and physical therapy, other patients might need more intensive procedures [99, 101, 102]. Hence, developing a unique approach that works for all patients is challenging [103]. Diagnostic injections play a fundamental part in confirming SIJ pain, while imaging techniques such as MRI and CT scans can help identify structural abnormalities and guide treatment decisions [26, 32]. Different studies support several strategies depending on their appreciation of the available data, clinical experience, and present state of knowledge [90, 104].

The management of SIJ pain evolves continuously. Biologics have been employed in managing inflammatory sacroiliitis linked to spondyloarthropathies in the past few years, but long-term consequences are still unknown [105–107]. Non-surgical management consists primarily of physical therapy to reduce pain, decrease stress on the SIJ, and improve function: joint manipulation [40, 53, 108], stabilisation exercises focused on strengthening the core, gluteal, and pelvic floor muscles [109], and stretching the lower back, hamstrings, and hip flexors [110]. Fascial manipulation concentrates on releasing tension in the fascia. In a pilot study evaluating the effectiveness of a single session of fascial manipulation at a distance from the painful region, significant pain reduction was evidenced [53]. Another randomised controlled trial [50] demonstrated the synergistic benefits of integrating motor control exercises and balance training in improving pain and functional outcomes. Lifestyle modifications, consisting of avoiding activities which might aggravate symptoms, preserving a low body weight, and using devices such as SI belts or braces, might decrease stress and pain [111].

Several drugs are also used to reduce inflammation and pain at the SIJ. Muscle relaxants, NSAIDs, analgesics (such as acetaminophen), and corticosteroids administered systemically or locally [2, 14, 112–116] can improve the symptoms efficiently or temporarily [33]. Other non-invasive strategies, such as image-guided injections of corticosteroids, prolotherapy (injection of a solution to prompt healing of ligaments and tendons around the joint), and radiofrequency nerve ablation, are used to relieve SIJ pain [2, 36, 72, 117–126]. Fluoroscopically guided SIJ injections are used to deliver medication directly into the joint. Injections typically contain a combination of a local anaesthetic and corticosteroids to reduce inflammation and provide pain relief. Chauhan et al. [57] compared the posteroanterior and classical oblique techniques for injections. Both methods were effective, but the posteroanterior approach had shorter fluoroscopy times, leading to lower radiation exposure and potentially lower costs. Autologous bone marrow mesenchymal concentrated aspirate has been employed to manage SIJ pain. In a randomised controlled trial on 80 patients, a one-time bone marrow concentrate aspirate injected into the SI joint significantly reduced pain and improved function within one year of follow-up [51]. Radiofrequency denervation involves radiofrequency energy to impair nerve function and has been extensively studied for SIJ pain [52, 54]. The lateral branch block is a minimally invasive procedure which implies injecting anaesthetic and anti-inflammatory medication near the lateral branches of the sacral nerves, potentially providing targeted pain relief. Cohen and Abdi [61] conducted a pilot study on 18 patients and stated that lateral branch blocks effectively managed SIJ pain. Navigation-assisted full-endoscopic radiofrequency rhizotomy is another recently introduced method of nerve ablation which has been advocated to promote superior precision and pain relief than other techniques [58]. Another variation of the conventional technique is cooled radiofrequency ablation, which uses a cooling mechanism to create larger lesions [62, 124]. A retrospective analysis of 88 patients revealed that cooled radiofrequency ablation did not provide superior pain relief compared to traditional methods [59]. However, a recent multicentre randomised study [62] found that cooled radiofrequency ablation provided superior pain relief and functional improvement compared to standard medical management.

Minimally invasive SIJ fusion is usually considered when conservative actions have failed [127, 128]. Surgical fusion stabilises the joint to reduce pain, and it is generally considered for patients not responsive to conservative management. Minimally invasive SIJ fusion using triangular titanium implants was more effective than conservative management in relieving pain and improving function and quality of life [77, 87]. Concerning surgical access, both posterior oblique and lateral exposures report similar improvements in function; however, the oblique technique was associated with greater pain reduction [56].

The relative efficacy of the treatments discussed in the present investigation differ broadly among researchers, and it is very challenging to define the best approach, its indication, and its value [101, 111]. Aspects such as effectiveness, duration of relief, and complications of SIJ pain management vary broadly among the approaches [101, 111] (Table 2).

**Table 2:** Efficacy, duration of relief, and complication risk of different approaches for the SIJ pain management

Type of management	Efficacy	Duration of relief	Complication risk
Physical therapy	Moderate	Medium term	Low
NSAIDs/pharmacologic therapy	Low–moderate	Short term	Low
Image-guided injections	Moderate–high	Short–medium term	Low–moderate
Radiofrequency denervation	High	Medium–long term	Moderate
Minimally invasive fusion	High	Long term	Moderate–high

Given the heterogeneous data and the lack of evidence on managing SIJ pain, recommendations for clinical practice are challenging. Management can be established concerning the type of pain and response to different modalities (from the less invasive to the most invasive). Physiotherapy, lifestyle modifications, and NSAIDs are the first-line treatments for patients with acute mild to moderate pain [50, 95, 129–132]. Patients with persistent SIJ pain, irresponsive to first-line conservative management, image-guided injections, and radiofrequency denervation, can be considered [122, 125]. Surgical SIJ fusion should be deserved for patients with severe or refractory pain who did not benefit from other less invasive interventions [104, 127, 128].

Many studies reported small sample sizes and limited length of the follow-up [50, 51, 55, 81]. These aspects negatively impact the generalisability of the reported results. Likewise, heterogeneity in study designs, different inclusion criteria, and outcome measures are evident. The variability in methods and protocols for radiofrequency ablation [52, 58] makes assessing and comparing the efficacy among techniques difficult. Additionally, the short-term follow-up periods of many studies [53, 62, 71] impair the capability to detect long-term outcomes and complications. Another limitation is the absence of placebo-controlled trials, which may be crucial for reducing bias and establishing connections [75]. Moreover, the subjective nature of pain assessment and the potential for placebo effects emphasise the necessity of more objective measures [61]. Previous trauma or intervention and general health were often not reported. Moreover, there is a lack of investigation comparing the efficacy of different conservative managements. Non-surgical management is fundamental in managing SIJ pain; additional research is needed. Given the high heterogeneity in endpoints, follow-ups, techniques, peri-procedure protocols, and rehabilitation procedures, additional statistical evaluations, although possible, have not been conducted. Finally, the present study was not registered prospectively, which might increase the risk of bias.

Future high-quality research and randomised controlled trials with longer follow-ups and greater sample sizes are necessary to promote more exhaustive evidence on the efficacy of different diagnostic and treatment methodologies and to fill the lack of information remaining in the literature. Future research should define explicit diagnostic algorithms, preferably using advanced imaging techniques; they should also identify robust therapeutic algorithms. Further comparisons between techniques, such as radiofrequency ablations or surgical techniques, are necessary. Examining the cooperative effects of combined conservative therapies and lifestyle modification may yield promising results. The regenerative medicine approach can play a crucial role in the future. Future studies should overcome the limitations of the existing evidence, offering clearer evidence-based guidelines for clinicians and surgeons and elaborating patient-tailored evidence-based algorithms for the management of SIJ pain.

## Conclusions

SIJ pain arises from multiple factors, requiring a multidisciplinary treatment approach. Non-surgical options primarily focus on physical therapy to relieve discomfort. Different medications aim to decrease inflammation and pain at the SIJ. Fluoroscopically guided SIJ injections allow for directly administering steroids or mesenchymal stem cells into the joint. Radiofrequency denervation is frequently used to address SIJ pain, while surgical fusion is usually reserved for cases where conservative treatment is ineffective. Future research should target the current evidence’s limitations, offering clearer, evidence-based guidelines for healthcare providers and developing patient-specific algorithms for SIJ pain management.

## Supplementary Information

Below is the link to the electronic supplementary material.

Supplementary file1 (DOCX 20 KB)

## Data Availability

The datasets generated during and/or analysed during the current study are available throughout the manuscript.
