# Supplementary material for: Management of sacroiliac joint pain: current concepts
Source: Eur J Orthop Surg Traumatol. 2025 May 21;35(1):208. doi: 10.1007/s00590-025-04308-2 (PMC12095441; doi:10.1007/s00590-025-04308-2)
Supplement: Supplementary file 1 — Supplementary file1 (DOCX 20 KB) [file 590_2025_4308_MOESM1_ESM.docx]

**Research Question:**

**Management of Sacroiliac Joint**

**Concept 1: Condition**

**Keywords:**

Back Pain

chronic low back pain

CHRONIC PAIN

Low back pain

pain

Pelvic girdle pain

Posterior sacroiliac complex

sacroiliac

Sacroiliac (SI) joint

sacroiliac joint

sacroiliac joint dysfunction

Sacroiliac joint pain

sacroiliac joint syndrome

Sacroiliac Joint Syndrome (SIJS)

sacroiliac pain

**Mesh:**

"Chronic Pain"[Mesh]

"Low Back Pain"[Mesh]

"Low Back Pain / pathology"[Mesh]

"Low Back Pain / physiopathology"[Mesh]

"Sacroiliac Joint"[Mesh]

"Sacroiliac Joint / pathology"[Mesh]

"Sacroiliac Joint / physiology"[Mesh]

"Sacroiliac Joint / physiopathology"[Mesh]

**Concept 2: Therapy**

**Keywords:**

ablation

Anesthesia, Local

Axial low back pain

bone marrow mesenchymal stem cells

Conservative management

Conservative pain management

conventional radiofrequency

cooled radiofrequency

cooled radiofrequency ablation

facet joint

Fluoroscopy

Injections

intra-articular

Intra-articular injections

minimally invasive spine surgery

Pain Management

pulsed radiofrequency

Radiofrequency

Radiofrequency ablation

Radiofrequency denervation

Sacroiliac joint denervation

Sacroiliac joint injection

Simplicity probe.

steroid injection

**Mesh:**

"Ablation Techniques"[Mesh]

"Analgesia / methods"[Mesh]

"Analgesics, Opioid"[Mesh]

"Arthrodesis / methods"[Mesh]

"Catheter Ablation"[Mesh]

"Catheter Ablation / methods"[Mesh]

"Conservative Treatment"[Mesh]

"Denervation"[Mesh]

"Denervation / instrumentation"[Mesh]

"Hyaluronic Acid / therapeutic use"[Mesh]

"Injections, Intra-Articular"[Mesh]

"Low Back Pain / drug therapy"[Mesh]

"Low Back Pain / surgery"[Mesh]

"Mesenchymal Stem Cells"[Mesh]

"Minimally Invasive Surgical Procedures"[Mesh]

"Nerve Block"[Mesh]

"Pain Management"[Mesh]

"Pulsed Radiofrequency Treatment / methods"[Mesh]

"Radiofrequency Ablation"[Mesh]

"Radiofrequency Therapy"[Mesh]

"Sacroiliitis / surgery"[Mesh]

"Steroids / therapeutic use"[Mesh]

**Concept 3: Interest of the outcome PROMS**

**Keywords:**

Quality of life

Visual Analog Scale (VAS)

VNS

**Mesh:**

"Pain Measurement"[Mesh]

"Patient Satisfaction"[Mesh]

"Postoperative Complications"[Mesh]

"Quality of Life"[Mesh]

"Treatment Failure"[Mesh]

"Treatment Outcome"[Mesh]

"Visual Analog Scale"[Mesh]

**Searching Strategy**

"Chronic Pain"[Mesh] OR "Low Back Pain"[Mesh] OR "Low Back Pain / pathology"[Mesh] OR "Low Back Pain / physiopathology"[Mesh] OR "Sacroiliac Joint"[Mesh] OR "Sacroiliac Joint / pathology"[Mesh] OR "Sacroiliac Joint / physiology"[Mesh] OR "Sacroiliac Joint / physiopathology"[Mesh] OR Back Pain OR chronic low back pain OR CHRONIC PAIN OR Low back pain OR pain OR Pelvic girdle pain OR Posterior sacroiliac complex OR sacroiliac OR Sacroiliac (SI) joint OR sacroiliac joint OR sacroiliac joint dysfunction OR Sacroiliac joint pain OR sacroiliac joint syndrome OR Sacroiliac Joint Syndrome (SIJS) OR sacroiliac pain

**AND**

"Ablation Techniques"[Mesh] OR "Analgesia / methods"[Mesh] OR "Analgesics, Opioid"[Mesh] OR "Arthrodesis / methods"[Mesh] OR "Catheter Ablation"[Mesh] OR "Catheter Ablation / methods"[Mesh] OR "Conservative Treatment"[Mesh] OR "Denervation"[Mesh] OR "Denervation / instrumentation"[Mesh] OR "Hyaluronic Acid / therapeutic use"[Mesh] OR "Injections, Intra-Articular"[Mesh] OR "Low Back Pain / drug therapy"[Mesh] OR "Low Back Pain / surgery"[Mesh] OR "Mesenchymal Stem Cells"[Mesh] OR "Minimally Invasive Surgical Procedures"[Mesh] OR "Nerve Block"[Mesh] OR "Pain Management"[Mesh] OR "Pulsed Radiofrequency Treatment / methods"[Mesh] OR "Radiofrequency Ablation"[Mesh] OR "Radiofrequency Therapy"[Mesh] OR "Sacroiliitis / surgery"[Mesh] OR "Steroids / therapeutic use"[Mesh] OR ablation OR Anesthesia, Local OR Axial low back pain OR bone marrow mesenchymal stem cells OR Conservative management OR Conservative pain management OR conventional radiofrequency OR cooled radiofrequency OR cooled radiofrequency ablation OR facet joint OR Fluoroscopy OR Injections OR intra-articular OR Intra-articular injections OR minimally invasive spine surgery OR Pain Management OR pulsed radiofrequency OR Radiofrequency OR Radiofrequency ablation OR Radiofrequency denervation OR Sacroiliac joint denervation OR Sacroiliac joint injection OR Simplicity probe. OR steroid injection

**AND**

"Pain Measurement"[Mesh] OR "Patient Satisfaction"[Mesh] OR "Postoperative Complications"[Mesh] OR "Quality of Life"[Mesh] OR "Treatment Failure"[Mesh] OR "Treatment Outcome"[Mesh] OR "Visual Analog Scale"[Mesh] OR Quality of life OR Visual Analog Scale (VAS) OR VNS

**SUMMARY**

("Chronic Pain"[Mesh] OR "Low Back Pain"[Mesh] OR "Low Back Pain / pathology"[Mesh] OR "Low Back Pain / physiopathology"[Mesh] OR "Sacroiliac Joint"[Mesh] OR "Sacroiliac Joint / pathology"[Mesh] OR "Sacroiliac Joint / physiology"[Mesh] OR "Sacroiliac Joint / physiopathology"[Mesh] OR Back Pain OR chronic low back pain OR CHRONIC PAIN OR Low back pain OR pain OR Pelvic girdle pain OR Posterior sacroiliac complex OR sacroiliac OR Sacroiliac (SI) joint OR sacroiliac joint OR sacroiliac joint dysfunction OR Sacroiliac joint pain OR sacroiliac joint syndrome OR Sacroiliac Joint Syndrome (SIJS) OR sacroiliac pain) AND ("Ablation Techniques"[Mesh] OR "Analgesia / methods"[Mesh] OR "Analgesics, Opioid"[Mesh] OR "Arthrodesis / methods"[Mesh] OR "Catheter Ablation"[Mesh] OR "Catheter Ablation / methods"[Mesh] OR "Conservative Treatment"[Mesh] OR "Denervation"[Mesh] OR "Denervation / instrumentation"[Mesh] OR "Hyaluronic Acid / therapeutic use"[Mesh] OR "Injections, Intra-Articular"[Mesh] OR "Low Back Pain / drug therapy"[Mesh] OR "Low Back Pain / surgery"[Mesh] OR "Mesenchymal Stem Cells"[Mesh] OR "Minimally Invasive Surgical Procedures"[Mesh] OR "Nerve Block"[Mesh] OR "Pain Management"[Mesh] OR "Pulsed Radiofrequency Treatment / methods"[Mesh] OR "Radiofrequency Ablation"[Mesh] OR "Radiofrequency Therapy"[Mesh] OR "Sacroiliitis / surgery"[Mesh] OR "Steroids / therapeutic use"[Mesh] OR ablation OR Anesthesia, Local OR Axial low back pain OR bone marrow mesenchymal stem cells OR Conservative management OR Conservative pain management OR conventional radiofrequency OR cooled radiofrequency OR cooled radiofrequency ablation OR facet joint OR Fluoroscopy OR Injections OR intra-articular OR Intra-articular injections OR minimally invasive spine surgery OR Pain Management OR pulsed radiofrequency OR Radiofrequency OR Radiofrequency ablation OR Radiofrequency denervation OR Sacroiliac joint denervation OR Sacroiliac joint injection OR Simplicity probe. OR steroid injection) AND ("Pain Measurement"[Mesh] OR "Patient Satisfaction"[Mesh] OR "Postoperative Complications"[Mesh] OR "Quality of Life"[Mesh] OR "Treatment Failure"[Mesh] OR "Treatment Outcome"[Mesh] OR "Visual Analog Scale"[Mesh] OR Quality of life OR Visual Analog Scale (VAS) OR VNS)
